# Genome atlas analysis based profiling of Akt pathway genes in the early and advanced human prostate cancer

**DOI:** 10.18632/oncoscience.482

**Published:** 2019-07-02

**Authors:** Abdulrahman Alwhaibi, Ravindra Kolhe, Fei Gao, Ewan K. Cobran, Payaningal R. Somanath

**Affiliations:** ^1^ Clinical and Experimental Therapeutics, College of Pharmacy, University of Georgia and Charlie Norwood VA Medical Center, Augusta, GA 30912; ^2^ Department of Pathology, Augusta University, Augusta, GA 30912; ^3^ Department of Clinical and Administrative Pharmacy, College of Pharmacy, University of Georgia, Athens, GA 30602; ^4^ Department of Medicine, Vascular Biology Center and Cancer Center, Augusta University, Augusta, GA 30912

**Keywords:** Akt1, Akt2, Akt3, cBioportal, TCGA, prostate cancer

## Abstract

Recent studies conducted in the mouse and cellular models suggest a stage-specific, differential effect of Akt activity modulation on tumor growth and metastasis in various cancers. In prostate cancer (PCa), although the deletion of Akt1 gene in a neuroendocrine model of TRansgenic Adenocarcinoma of the Mouse Prostate (TRAMP) blunted oncogenic transformation and tumor growth, Akt1 suppression in the advanced PCa resulted in the activation of transforming growth factor-β pathway and enhanced metastasis to the lungs. Such a dual role for the Akt isoforms and its signaling partners has not been investigated in human PCa. In the current study, we performed genomic database analysis of Akt isoforms and associated pathway molecules in human prostate adenocarcinoma, castration-resistant PCa, neuroendocrine PCa and metastatic PCa for mutations, genetic alterations, mRNA and protein expressions and activating phosphorylations from cBioportal. Results from the protein data analysis from the cBioportal were compared to the results of our data on human PCa tissue analysis and the cellular effects of Akt1 suppression using MK-2206 on PCa cell aggressiveness. Our study indicates the existence of a dual role for Akt1 in PCa and warrants a large-scale analysis of the early and advanced stage PCa clinical samples for further clarity.

## INTRODUCTION

Metastatic prostate cancer (PCa) is the leading cause of cancer-related deaths in men in the US and Europe [1]. Although slow-growing cancer, PCa that has metastasized to the bone, lungs, and brain becomes difficult to treat [2]. Uncertainties in the molecular mechanisms leading to the switch from early to advanced PCa are the underlying reason for the unreliable screening measures and ineffective treatments in the management of early and metastatic PCa [3].

Phosphoinositide-3-Kinase (PI3K)/Akt pathway has a well-established role in the regulation of cellular processes essential for cell survival such as metabolism, proliferation, growth, anti-apoptosis and cytoskeletal reorganization [4]. Aberrant activation of the PI3K/Akt pathway has been recognized as an essential step towards the initiation and progression of many cancers [5]. Activation of this pathway is driven by genetic mutation or activity deregulation of the upstream components such as receptor tyrosine kinases (RTKs) [6], non-RTKs such as Src family kinases [7] or modulation of the downstream components including PTEN inactivation or deletion, PI3K constitutive activation or amplification, Akt hyperactivation and other genetic changes in signaling molecules involved in this pathway [8].

Although Akt pathway has been targeted for cancer therapy for many years, as of today no drugs that target Akt has been approved for any cancer treatments. Recently we showed that Akt1, the predominant Akt isoform in the PCa cells [9] and tumor vascular cells [10-12] plays a dual, reciprocal role in prostate tumor growth and metastasis [13]. Such a finding has also been reported in three other cancer types such as the breast [14, 15], liver [16] and non-small cell lung cancer [17]. Interestingly, our most recent study has indicated the important role of several microRNAs in the dual, stage-specific role of Akt1 in cancer with Akt1 activity suppression in the early and advanced stages of murine neuroendocrine model of PCa in a Transgenic adenocarcinoma of the mouse prostate (TRAMP) tissues resulting in entirely different set of microRNA expression [18]. Further, a more recent study from our laboratory has demonstrated that endothelial-specific loss of Akt1 in mice promotes PCa metastasis to the lungs [19]. These preclinical studies have identified Akt1 as a molecule that promotes tumor growth but inhibits metastasis in cancer. The above studies also have identified a reciprocal link between Akt1 and TGFβ pathways in promoting cancer cell epithelial-to-mesenchymal transition (EMT) and metastasis. As of today, the genomic and proteomic changes in Akt isoforms and their signaling pathway molecules in the primary and advanced stages of human PCa have not been studied in detail.

In the current study, we performed a genomic and proteomic database analysis (http://www.cbioportal.org) [20, 21] of Akt pathway from human PCa patient studies performed in various types such as the human prostate adenocarcinomas [22-24], castration-resistant PCa [25], neuroendocrine PCa [26] and metastatic PCa [25, 27], and determined the alterations in mRNA, protein expression, and genetic mutations. There were data from a total of 13 studies available in cBioportal performed on PCa patient samples, and 6 of them have the data on mRNA and one has proteomic expression changes in various genes. These include a study on primary PCa, two studies in prostatic adenocarcinoma (both primary and metastatic), a study on neuroendocrine PCa and two studies on castration-resistant metastatic PCa. Genomic data mining analyses from these studies available on cBioportal with respect to the alterations in the Akt pathway molecules are presented in this article. Our results strongly suggest the existence of a dual role for the Akt pathway in the early and advanced PCa and warrants large-scale analysis of PCa patient samples for further clarity on this new information.

## RESULTS

### cBioportal cancer genome atlas show minimal mutations in the Akt isoforms in PCa

We first determined the existence of any known gene mutations in the 3 Akt isoforms. Our analysis of the six PCa studies from the cBioportal genome atlas indicated no significant genetic mutations in any of the Akt isoforms that compromised its activity. A very small population of PCa patients exhibited a single mutation in the Akt1 isoform resulting in E17K alteration (Supplemental Figure 1). While this was identified to be a missense mutation, similar mutations in the Akt2 and Akt3 isoforms resulted in the alterations of A214V and A101G residues (Supplemental Figure 1), once again in a very small population of PCa patients. Together, these studies indicated that mutations in the Akt isoforms are not major determinants for its activity deregulation contributing to the onset or aggressiveness of PCa.

### Analysis of the integrative genomic profiling of human primary tumors and metastatic PCa by the MSKCC identifies alterations in the Akt pathway genes

**FIGURE 1:**
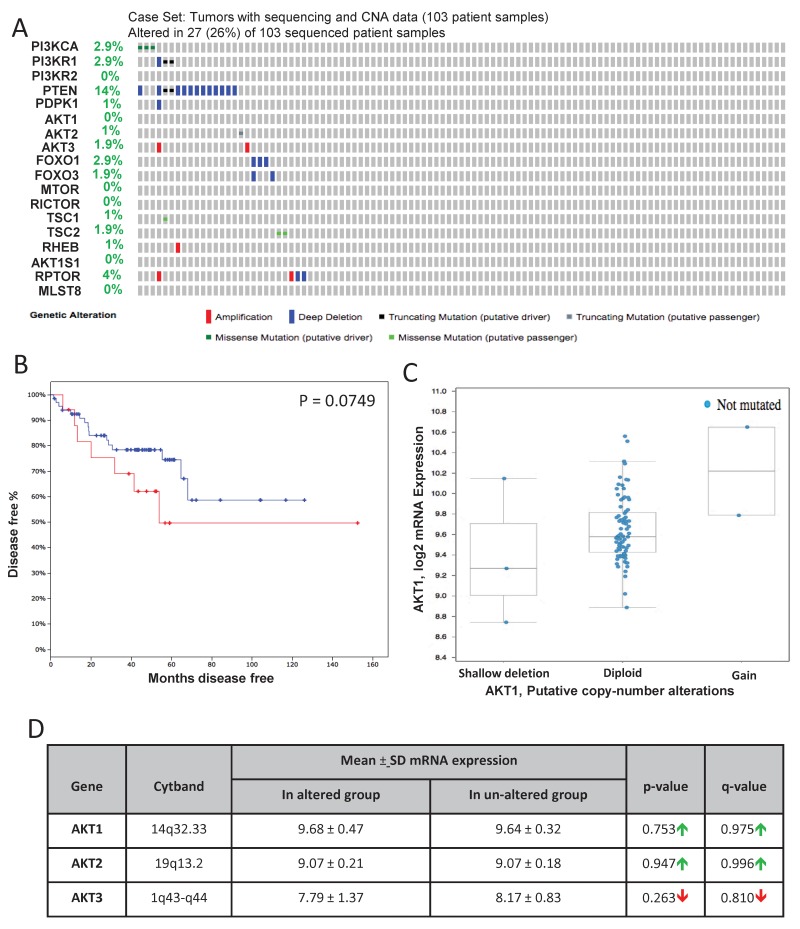
Gene alterations in Akt pathway human prostate adenocarcinoma **(A)** Oncoprint showing genomic alterations in the Akt pathway genes in human prostate adenocarcinoma samples based on the integrative genomic profiling performed by the MSKCC study (Taylor BS et al, Cancer Cell, 2010). **(B)** Kaplan–Meier survival analysis showing decreased disease-free survival in patients with observed Akt pathway alterations (indicated in red) compared to the unaltered group (indicated in blue). **(C)** A plot showing the relationship between Akt1 mRNA abundance and copy-number alteration (CAN) in the Akt1 gene in human prostate adenocarcinoma. **(D)** Chart showing the overall mRNA expression of Akt1, Akt2, and Akt3 in the prostatic adenocarcinoma tissues with observed Akt pathway alterations compared to the un-altered group.

Genomic profiling by the MSKCC group is one of the first among the large-scale analysis of genes performed in the primary and advanced PCa patient samples [22]. Out of the 216 patient samples used in the analysis, genomic data on 103 patient samples and 149 control samples are available on cBioportal. In our analysis, approximately 26% (27 out of 103) of the patients exhibited alterations in genes from the PI3K/Akt pathway (Figure 1A) leading to reduced disease-free survival in patients (Figure 1B). Among these, 14 % of the alterations were due to a deep deletion in the PTEN gene (Figure 1A). Whereas 2.9% of the patients exhibited missense mutations (putative driver) in the PI3K catalytic subunit, 2.9% of the patient samples showed a deletion or truncating mutation (putative driver) in the PI3K regulatory subunit-1 (Figure 1A). Deep deletions were also observed in FoxO1 and FoxO3 in 2.9% and 1.9% of the patient population, respectively (Figure 1A). Interestingly, no genetic alterations were observed in the Akt isoforms except amplification of Akt3 in two patients (Figure 1A). Despite the markedly increased Akt1 mRNA in the screened tumors, the majority did not have amplified the Akt1 gene as shown by a diploid Akt1 (Figure 1C). There were also no significant differences in the mean mRNA expression levels of Akt1, Akt2 and Akt3 isoforms between the altered and un-altered groups (Figure 1D). Together, our analysis indicated that while alterations in many Akt pathway components such as PTEN may contribute to the hyperactivation of Akt isoforms, the evidence on the direct effect of enomic alteration on their activity is unclear.

### Genomic analysis of the human neuroendocrine PCa samples by the Trento/Broad/Cornell study reveals high alterations in the Akt pathway genes

**FIGURE 2:**
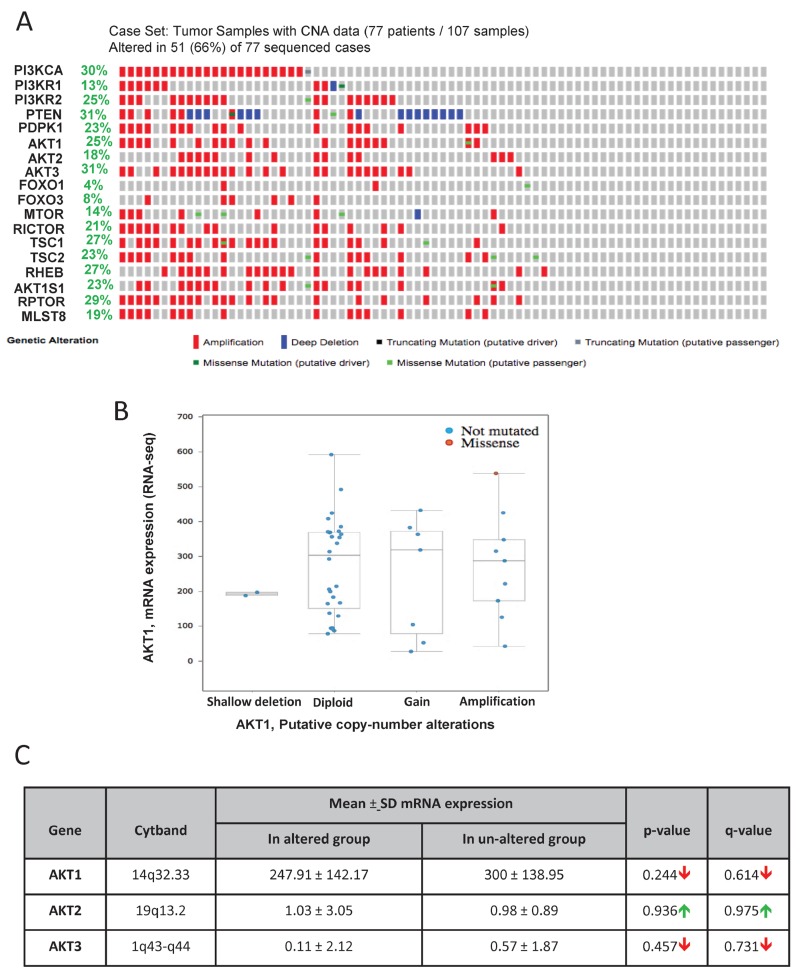
Gene alterations in Akt pathway in castration-resistant neuroendocrine PCa **(A)** Oncoprint showing genomic alterations in the Akt pathway genes in human castration-resistant neuroendocrine PCa samples based on the genomic profiling by the Trento/Cornell/Broad study (Beltran H et al, Nat Med, 2016). **(B)** A plot showing the relationship between Akt1 mRNA abundance and copy-number alteration (CAN) in the Akt1 gene in human castration-resistant neuroendocrine PCa. **(C)** Chart showing the overall mRNA expression of Akt1, Akt2, and Akt3 in neuroendocrine PCa samples with observed Akt pathway alterations compared to the un-altered group.

A genomic study by the Trento/Broad/Cornell group was primarily focused on the human neuroendocrine PCa [26]. Out of the 107 patient samples used for the analysis, genomic data on 77 patient samples is available on cBioportal. In our analysis, approximately 66% (51 out of 77) of the patient samples showed genetic alterations in genes from the PI3K/Akt pathway (Figure 2A). The highest level of genetic alterations was observed in the case of PTEN deletion or amplification (31%). High amplification of genes such as PI3K catalytic subunit (30%), PI3K regulatory subunit-1 (13%), PI3K regulatory subunit-2 (25%), and the Akt isoforms (25%, 18% and 31% in Akt1, Akt2, and Akt3, respectively) were also observed (Figure 2A). Gene amplifications were observed in the mTOR pathway genes such as mTOR (14%), Raptor (29%), Rictor (21%), Tuberous sclerosis complex-1 (TSC1; 27%), TSC2 (23%) and Rheb (27%) (Figure 2A). Data from the RNA-seq analysis indicated gain and amplification of Akt1 mRNA in a large population of these patients (Figure 2B). Interestingly, there were no significant differences in the mean mRNA expression levels of Akt1, Akt2 and Akt3 isoforms between the altered and un-altered groups (Figure 2C). Together, our analysis indicated that although deletion of PTEN and amplification many other Akt pathway genes were observed in neuroendocrine PCa samples, there was no significant difference in the mean mRNA levels of the Akt isoforms between the altered group of patients vs. the un-altered group.

### Exome sequence analysis of the human prostate adenocarcinoma samples by the Broad/Cornell study shows very low alterations in the Akt pathway genes

**FIGURE 3:**
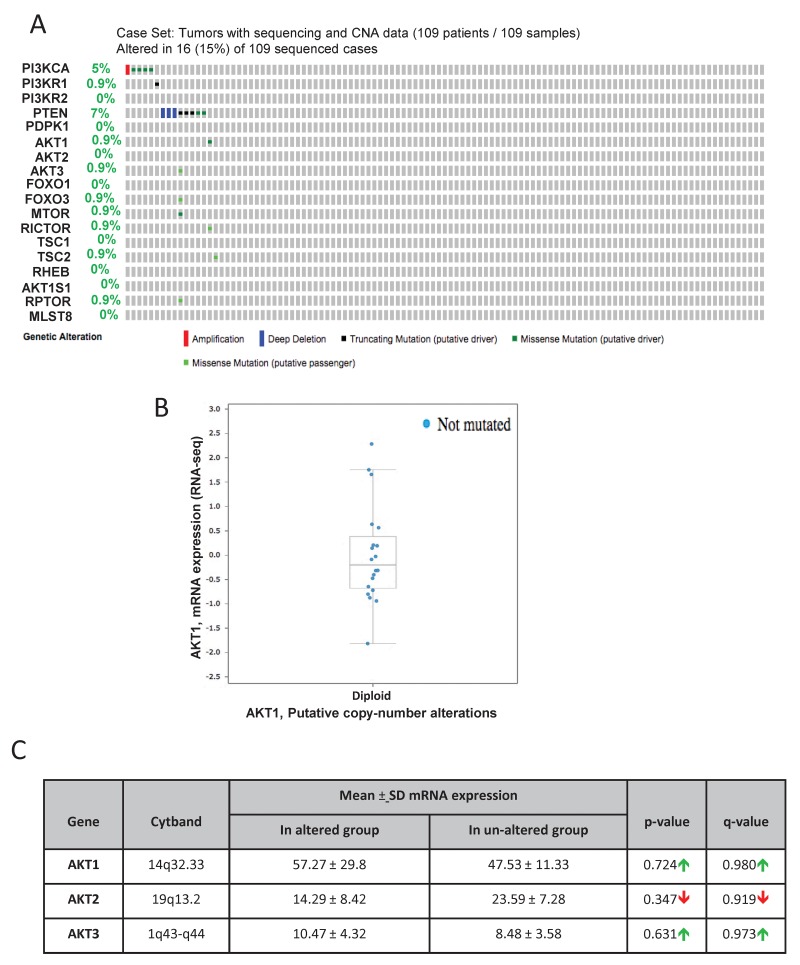
Gene alterations in Akt pathway in human non-metastatic prostate adenocarcinoma **(A)** Oncoprint showing genomic alterations in the Akt pathway genes in human non-metastatic prostate adenocarcinoma tissues based on the exome sequencing performed by the Broad/Cornell study (Barbieri CE et al, Nat Gen, 2012). **(B)** A plot showing the relationship between Akt1 mRNA abundance and copy-number alteration (CAN) in the Akt1 gene in human non-metastatic prostate adenocarcinoma. **(C)** Chart showing the mean mRNA expression of Akt1, Akt2, and Akt3 in the non-metastatic prostatic adenocarcinoma tissues with observed Akt pathway alterations compared to the un-altered group.

Exome sequencing by the Broad/Cornell group was primarily focused on the human prostate adenocarcinoma [23]. Genomic data on all the 109 sequenced patient samples are available on cBioportal. In our analysis, only 15% (16 out of 109) of the patient samples showed genetic alterations in genes from the PI3K/Akt pathway (Figure 3A). Like in the other studies, alterations were primarily observed in PTEN (7%) in the form of gene deletion or loss of function mutations (Figure 3A). Although amplification in the PI3K catalytic subunit was found in a single patient, single cases of missense mutations were also observed in PI3K catalytic subunit, Akt1, Akt3, FoxO3, mTOR, Rictor, TSC2, and Raptor (Figure 3A). Data from the RNA-seq analysis indicated no significant gain or amplification of Akt1 mRNA in these prostate adenocarcinoma samples (Figure 3B). Also, there were no significant differences in the mean mRNA expression levels of Akt1, Akt2 and Akt3 isoforms between the altered and un-altered groups (Figure 3C). Overall, this indicates no significant genetic alterations in the PI3K/Akt pathway genes among prostate adenocarcinoma patients.

### Clinical genomics of the human metastatic PCa reveals high alterations in the Akt pathway genes

**FIGURE 4:**
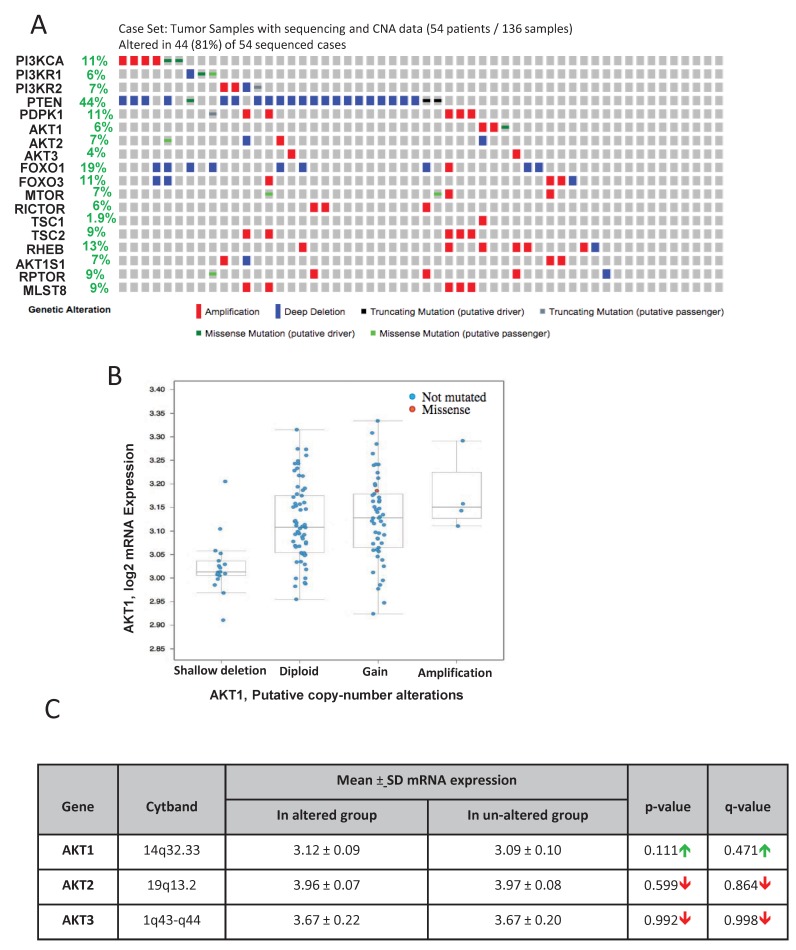
Gene alterations in Akt pathway in human metastatic PCa **(A)** Oncoprint showing genomic alterations in the Akt pathway genes in human metastatic PCa tissues based on the genomic analysis performed by the Fred Hutchinson study (Kumar A et al, Nat Med, 2016). **(B)** A plot showing the relationship between Akt1 mRNA abundance and copy-number alteration (CAN) in the Akt1 gene in human metastatic PCa. **(C)** Chart showing the mean mRNA expression of Akt1, Akt2 and Akt3 in the metastatic PCa tissues with observed Akt pathway alterations compared to the un-altered group.

Genome analysis by the Fred Hutchinson group was primarily focused on the human metastatic PCa [25]. Out of the 136 patient samples used for the analysis, genomic data on 54 patients is available on cBioportal. In our analysis, approximately 81% (44 out of 54) of the patient samples exhibited genetic alterations in genes from the PI3K/Akt pathway (Figure 4A). The highest level of genetic alterations was once again observed in the case of PTEN deletion or loss of function mutations (44%). Although high alterations of genes such PI3K catalytic subunit amplification (11%), FoxO1 gene deletion (19%) and Rheb amplification (13%) were also observed, only 4-7% alterations were noted in the Akt isoforms (Figure 4A). Data from the mRNA expression analysis indicated a significant gain of Akt1 mRNA in a large population of the metastatic PCa samples (Figure 4B). However, there were no significant differences in the mean mRNA expression levels of Akt1, Akt2 and Akt3 isoforms between the altered and un-altered groups (Figure 4C).

**FIGURE 5:**
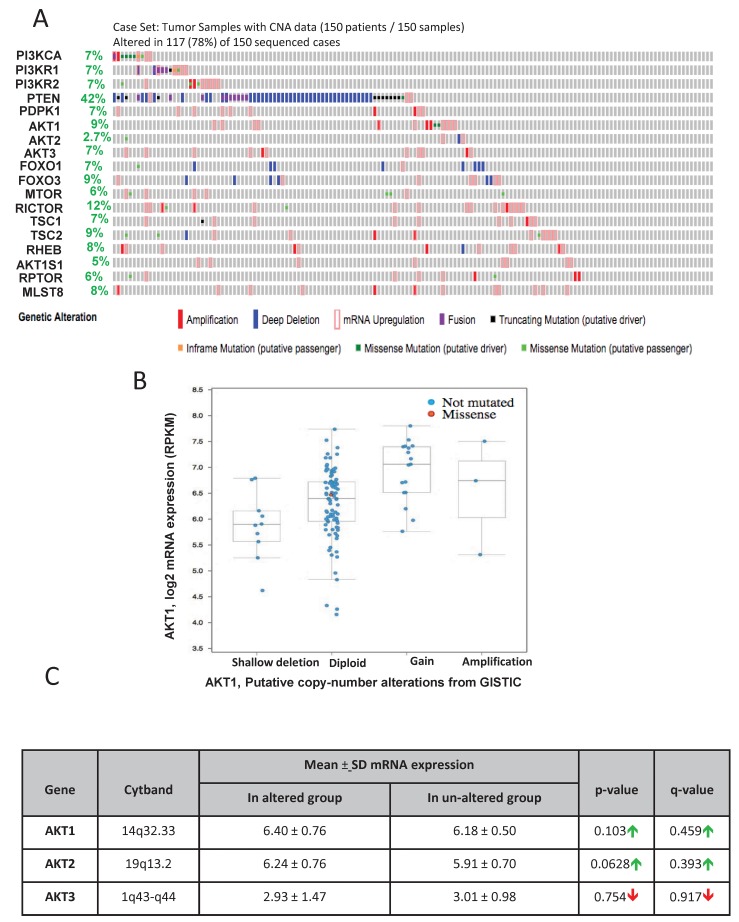
Gene alterations in Akt pathway in human advanced PCa **(A)** Oncoprint showing genomic alterations in the Akt pathway genes in human advanced (metastatic castration-resistant) PCa tissues based on the genomic analysis performed by the MSKCC/UMICH study (Robinson D et al, Cell, 2015). **(B)** A plot showing the relationship between Akt1 mRNA abundance and copy-number alteration (CAN) in the Akt1 gene in human advanced PCa. **(C)** Chart showing the mean mRNA expression of Akt1, Akt2 and Akt3 in the advanced PCa tissues with observed Akt pathway alterations compared to the un-altered group.

Another study on metastatic PCa was conducted by the MSKCC group [27]. Genomic data on all the 150 sequenced patient samples are available on cBioportal. In our analysis, approximately 78% (117 out of 150) of the patient samples indicated genetic alterations in genes from the PI3K/Akt pathway (Figure 5A). Similar to the Fred Hutchinson study, 42% of the alterations were found in the PTEN gene deletion, fusion or loss of function mutations (Figure 5A). Minimal alterations (2-12%) were also found in most other genes of the PI3K/Akt pathway in metastatic PCa samples (Figure 5A). Data from the mRNA expression analysis indicated some gain of Akt1 mRNA in a smaller population of the metastatic PCa samples (Figure 5B). However, there were no significant differences in the mean mRNA expression levels of Akt1, Akt2 and Akt3 isoforms between the altered and un-altered groups (Figure 5C). Together, these two studies indicate the importance of PTEN inactivation in the activation of the PI3K/Akt pathway in metastatic PCa.

### TCGA study of the primary PCa identifies genetic alterations in the Akt pathway genes

**FIGURE 6:**
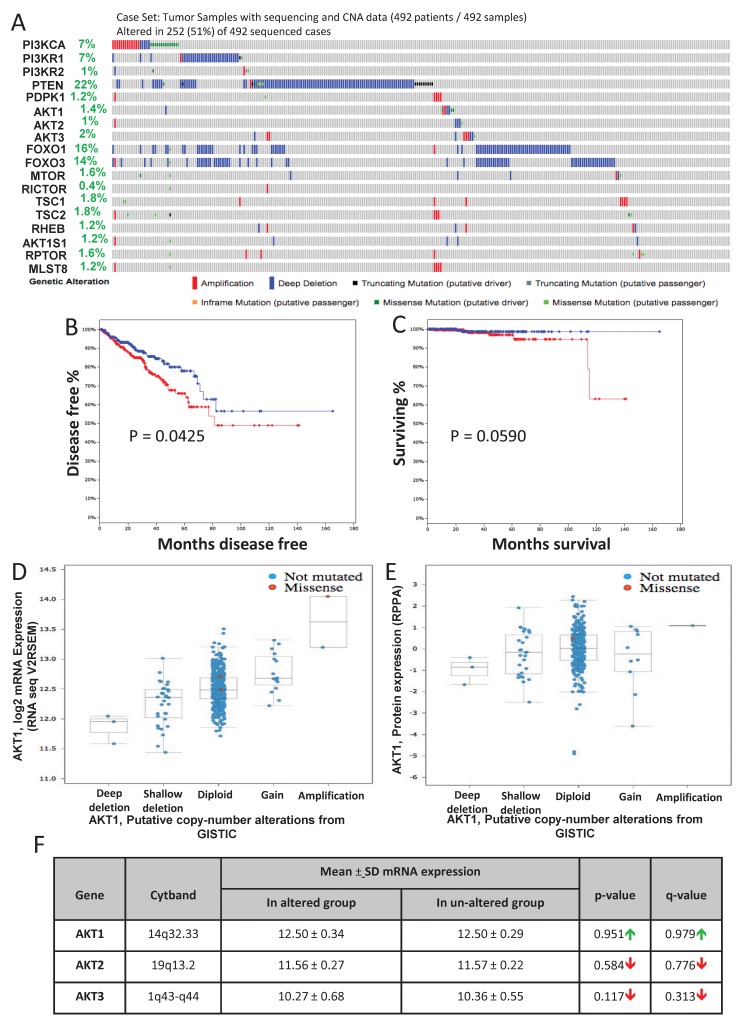
Gene alterations in Akt pathway in human primary prostate adenocarcinoma **(A)** Oncoprint showing genomic alterations in the Akt pathway genes in human primary (non-metastatic) PCa tissues based on the analysis by the cancer genome atlas research network (TCGA, Cell, 2015). **(B)** Kaplan–Meier survival analysis showing decreased disease-free survival in patients with observed Akt pathway alterations (in red) compared to the un-altered (in blue) group. **(C)** Kaplan–Meier survival analysis showing decreased overall survival in patients with observed Akt pathway alterations compared to the un-altered group. **(D)** A plot showing the relationship between Akt1 mRNA abundance and copy-number alteration (CAN) in the Akt1 gene in human primary PCa. **(E)** A plot showing the relationship between Akt1 protein expression and copy-number alteration (CAN) in the Akt1 gene in human primary PCa. **(F)** Chart showing the mean mRNA expression of Akt1, Akt2 and Akt3 in the PCa tissues with observed Akt pathway alterations compared to the un-altered group.

**FIGURE 7:**
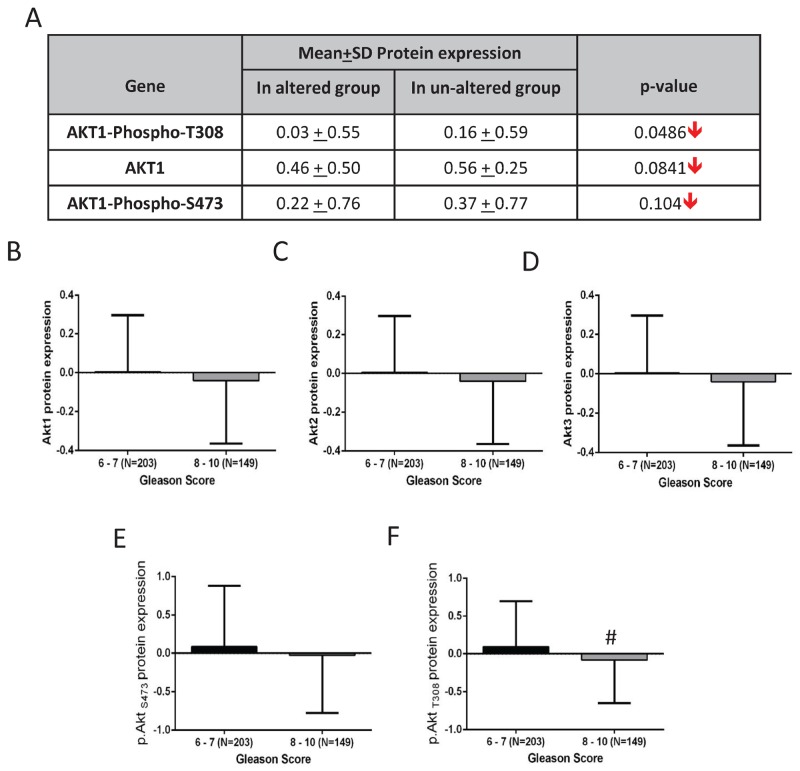
Alterations in Akt1 protein expression and its activity in human primary prostate adenocarcinoma **(A)** Chart showing a decline in the mean protein expression of Akt1, p.AktS473 and p.AktT308 in the PCa tissues with observed Akt pathway alterations compared to the un-altered group based on the analysis by the cancer genome atlas research network (TCGA, Cell, 2015). **(B-F)** Patients stratification based on Gleason score [higher (8-10) and lower (6-7) group] showed a declining trend in Akt1, Akt2, Akt3, p.AktS473 and a significant reduction in p.AktT308 protein/phosphorylation levels in the higher compared to the lower Gleason score group. #P < 0.01; Unpaired Student t-test for two group analysis (GraphPad Prism 6.01). Data are presented as means ± SD.

Genomic profiling of a large collection of primary PCa samples identified genetic alterations in the Akt pathway genes [24]. Genomic data on all the 492 sequenced patient samples are available on cBioportal and NCI TCGA sites. In our analysis, approximately 51% (252 out of 492) of the patient samples showed alterations in genes from the PI3K/Akt pathway (Figure 6A) leading to reduced disease-free survival (Figure 6B) and overall survival in patients (Figure 6C). Among these, 22 % of the alterations were due to a deep deletion in the PTEN gene, 16% in FoxO1 gene deletion, 14% were in FoxO3 gene deletion and 7% in the PI3K regulatory subunit-1 (Figure 6A). Interestingly, no genetic alterations were observed in the Akt isoforms except isolated cases of amplification or deletion in Akt1 (1.4%), Akt2 (1%) and Akt3 (2%) (Figure 6A). Despite the markedly increased Akt1 mRNA in the screened tumors, the majority did not have amplified the Akt1 gene as shown by a diploid Akt1 (Figure 6D). A significant difference in the protein expression of Akt1 was also not observed in these patients' samples (Figure 6E; Figure 7A-D).

### Suppression of Akt activity (reduced Akt phosphorylation) is linked to the promotion of EMT in the advanced stage PCa via increased expression of TGFβ1

We next reviewed the data from www.clinicaltrials.gov on the effects of Akt inhibitor MK-2206 in various cancer clinical trials. Our analysis indicated no significant clinical benefits of MK-2206 on many of the advanced stage, metastatic cancers (Supplemental Table 1). In many trials, particularly the metastatic cancers, MK-2206 treatment showed reduced overall survival and progression-free survival of the cancer patients (E.g. NCT01253447 and NCT01658943). Nevertheless, some benefits of MK-2206 treatment were observed in the early-stage tumors [28-30].

Since none of the 6 genomic studies showed any significant gain of Akt isoform mean mRNA and protein expression levels between the altered and un-altered groups, we next compared the levels of pAktSer473 and pAktThr308 phosphorylation levels (level of Akt activation) with Gleason score. Our analysis of data from the TCGA study (N=352 patients) showed significant in pAktT308 levels between low Gleason score (Score 6-7) versus high Gleason score (Score 8-10; N=149; N=203) samples (Figure 7E). Although not significant, there was a strong trend correlating increased pAktS473 levels between low Gleason score (Score 6-7) versus high Gleason score (Score 8-10) samples (Figure 7F).

**FIGURE 8:**
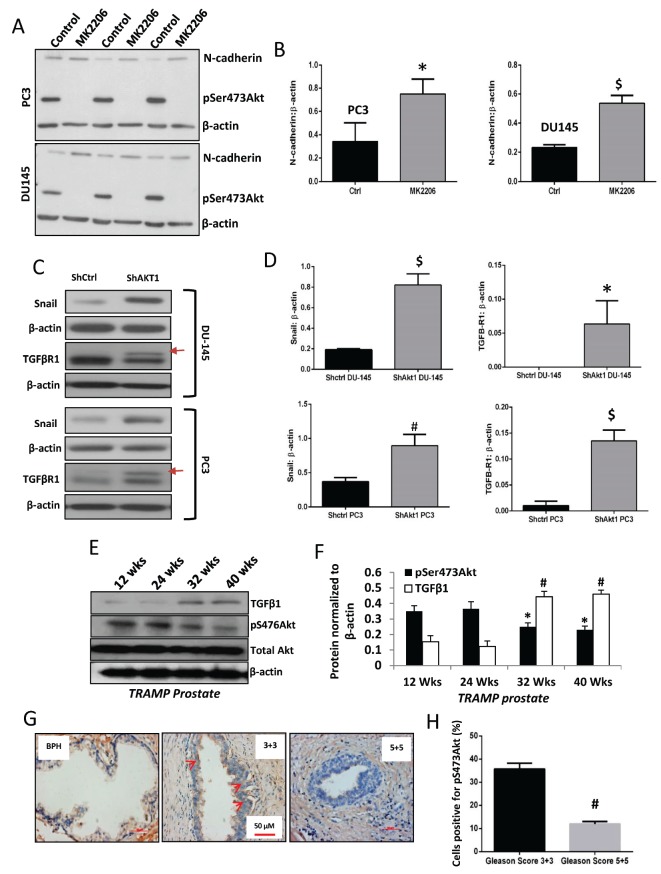
A decrease in Akt phosphorylation (activity), not expression is linked to EMT in PCa **(A)** Representative Western blot images of PC3 and DU145 cell lysates treated with DMSO (control) or Akt inhibitor MK2206 for 72 hours (5 μM) showing changes in the phosphorylation of Akt associated with changes in the expression of mesenchymal marker N-cadherin. **(B)** Bar graphs showing changes in N-cadherin expression in PC3 and DU145 cells with MK2206 treatment (n=3). **(C)** Representative Western blot images of PC3 and DU-145 ShControl and ShAkt1 cell lysates showing changes in the expression of TGFβ-R1 and mesenchymal transcription factor Snail. **(D)** Representative bar graph of band densitometry analysis for TGFβR1 and Snail1 of PC3 and DU-145 ShControl and ShAkt1 cell lysates (n=3). **(E-F)** Western blot images and band densitometry analysis of TRAMP prostate lysates collected from 12, 24, 32 and 40 wks-old mice, and analyzed for changes in pS473Akt and TGFβ1 expressions, showing an inverse relationship between pS473Akt (decreased) and TGFβ1 (increased) in the high-grade tumor (n=4). **(G-H)** Immunohistochemistry of early stage PCa (Gleason 3+3) showing a higher number of phosphorylated Akt (pSer473, active) positive cells compared to the advanced stage (Gleason 5+5) (n=5) as counted using Image-J software and percentage of pAkt-positive cells were determined. *P<0.01 compared to pS473Akt on 12 wks; #P>0.01 compared to TGFβ1 on 12 wks. $P < 0.01; Unpaired Student t-test for two groups analysis (GraphPad Prism 6.01). Data are presented as means ± SD.

In order to further explore this, we determined the effect of Akt activity suppression with MK2206 treatment on epithelial-to-mesenchymal transition (EMT) and aggressiveness of PC3 and DU145 human PCa cells. In our analysis, treatment with 5 μM MK2206 revealed reduced Akt phosphorylation associated with the increased expression of EMT marker N-cadherin in both PC3 and DU145 human PCa cell lines (Figure 8A-B). Similarly, Akt1 gene deletion using shRNA, hence reduction in its activity, also resulted in increased expression of EMT transcription factor Snail and TGFβ-R1 in PC3 and DU145 cells (Figure 8C-D). A stage-specific analysis of *TRAMP* prostates collected at 12, 24, 32 and 40 weeks indicated an inverse relationship between S473Akt phosphorylation (activity) and TGFβ1 expression (Figure 8E-F), where reduced Akt1 phosphorylation in the advanced PCa is associated with the increased TGFβ1. A similar effect was also observed in the staining of phosphorylated Akt (pSer473Akt) in human PCa tissues, where a decreased expression of pSer473Akt in high Gleason score (5+5) PCa tissues was observed compared to low Gleason score samples (3+3), particularly in the proliferating luminal cells (Figure 8G-H).

**FIGURE 9:**
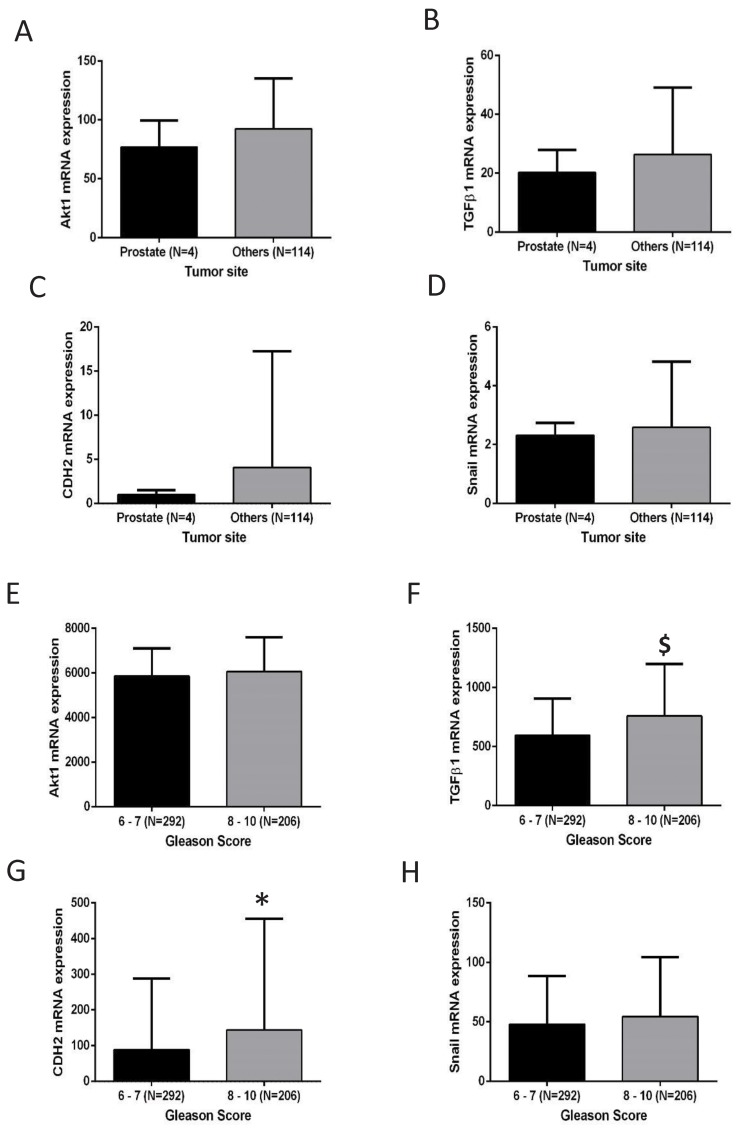
EMT is positively correlated to higher Gleason score and PCa metastasis with no change in the Akt1 expression **(A-D)** Castration-resistant PCa from patients in the MSKCC/UMICH study (Robinson D et al, Cell, 2015) stratified based on the anatomic site [prostate and others (bone, muscles, neck, chest wall, thoracic epidural, liver, bladder, penis, pelvis, lymph node, perirectal, retroperitoneum and soft tissue) group] showed no change in Akt1 mRNA level with a trend toward elevation of TGFβ1, CDH2 (N-cadherin) and Snail in the others compared to the prostate group. **(E-H)** Primary prostate adenocarcinoma from patients in the cancer genome atlas study (TCGA, Cell, 2015) stratified based on Gleason score [higher (8-10) and lower (6-7) group] showed no change in Akt1 mRNA level with a significant increase in TGFβ1 and CDH2 (N-cadherin) and a trend toward elevation of Snail in the higher compared to the lower Gleason score group. *P < 0.05; $P < 0.01; Unpaired Student t-test for two groups analysis (GraphPad Prism 6.01). Data are presented as means ± SD.

In order to illustrate the clinical implications of these results, mRNA data of patients from MSKCC/UMICH (Robinson D *et al*, Cell, 2015) study were used to determine a correlation between tumor anatomic site and EMT. Strikingly, although Akt1 was not significantly different between the selected cohorts, a trend towards increased TGFβ1, CDH2 (N-cadherin) and Snail were observed in the metastatic tumor sites (N=114) compared to the tumors localized in the prostate (N=4) (Figure 9A-D). Since Snail and TGFβ1 protein levels were not available in the TCGA data, we determined the mRNA levels of these genes and CDH2 (N-cadherin). Intriguingly, although not significant, there was a strong trend correlating increased TGFβ1, Snail and CDH2 levels in the higher (N=206) compared to the lower Gleason score group (N=292) (Figure 9E-H), indicating that the suppression of Akt1 activity in advanced PCa could promote EMT and metastasis.

## DISCUSSION

The serine/threonine kinase Akt has long been known for its role in cell survival and proliferation via modulation of its downstream substrates such as glycogen synthase kinase-3 (GSK3), FoxO, Bad and Bcl2, etc. [12] in promoting tumor growth [9, 13, 31-37]. Many laboratories have demonstrated that Akt isoforms are expressed and activated differentially in tumors [38, 39], thus the notion that they have distinct roles in cancer is well accepted. Intriguingly, the most recent studies *in vitro* and animal models [39-44] on the role of Akt in advanced cancers clearly demonstrate an unexpected, suppressive role of Akt in cancer metastasis. A previous study from our laboratory demonstrated that although Akt1 is essential for oncogenic transformation in a neuroendocrine PCa *TRAMP* mouse model, pharmacological inhibition of Akt using triciribine in advanced PCa bearing *TRAMP* mice or genetic ablation of Akt1 gene in PC3 and DU145 human PCa cells augmented EMT and metastasis [13]. However, such a negative correlation between Akt activity and metastasis has never been studied in human PCa. Hence, in the current study, we compared the genomic data on the Akt pathway genes based on six studies that have deposited their sequencing data in cBioportal. We also determined the effect of Akt activity suppression by MK-2206, a drug used in clinical trials for cancer, analyzed a small population of human PCa samples for the level of activating Akt phosphorylation in the advanced stage PCa compared to early stage and BPH tissues, and highlighted the correlation between Akt1 mRNA or protein expression/activity and EMT in the advanced PCa based on few selected CBioportal studies.

Initial reports on the inhibitory effects of Akt1 activation on cancer cell migration and invasion *in vitro* came from Toker laboratory [45]. In this study, siRNA-mediated Akt1 deletion promoted breast cancer cell invasion via the human homolog of the E3 ubiquitin ligase (HDM2)-mediated ubiquitination and degradation of the nuclear factor of activated T-cells (NFAT). Another study by the same group linked Akt1 activity to palladin, an actin-binding protein that anchors cytoskeletal proteins to actin fibers thus reducing reduced stress fiber formation and attenuating breast cancer cell invasion *in vitro* [46]. Subsequent studies by other laboratories further supported this theory by demonstrating enhanced ERK activation resulting in the loss of cuboidal-epithelial morphology in Akt1, but not Akt2-deficient MCF-10A cells, promoting EMT and invasion *in vitro* [47] leading to miR-200 abundance [14]. Akt1 silencing in either of the androgen-sensitive or androgen-resistant PCa cells induced β1-integrin activity and their localization in the cell periphery (in PC3 cell line) thus promoting focal adhesion formation and invasion [48]. Interestingly, although Akt1 overexpression in ERbB-2 transgenic mice resulted in the up-regulation of cyclin D1 levels accompanied by accelerated mammary tumorigenesis, tumors developed in these bitransgenic mice were less invasive to the surrounding tissues compared to the tumors in ERbB-2 strain [49]. More importantly, lung metastatic lesions were significantly less in the bitransgenic mice indicating that tumors developed with activated Akt1 had less metastatic properties compared to the ones with less active Akt1.

After a decade-long hiatus, there has been a renewed interest on the dual role of Akt in cancer after it was discovered that the deletion of Akt1 gene in Akt2^+/−^ mice potentiate inflammation-induced hepatic cancer [16]. Following this, Akt1 was identified as a negative regulator of breast cancer metastasis *in vivo* via proteolytic degradation of twist-1, a transcriptional factor that induces EMT [15]. Interestingly, inhibition of Akt by MK-2206 treatment in the above study led to twist-1 stabilization, promoting breast cancer cell invasion *in vitro* and lung metastasis *in vivo* accompanied by increased N-cadherin and vimentin, and decreased E-cadherin expressions. Latest in this series are the two parallel reports from NSCLC and PCa. Whereas Akt inhibition by MK-2206 *in vitro* promoted NSCLC invasion and metastasis through the activation of MARCKs-LAMC2 [17], Akt inhibition by triciribine promoted PCa EMT and metastasis in a neuroendocrine mouse model of TRAMP [13]. Furthermore, silencing of Akt1 in PCa cell lines (PC3 and DU145) enhanced EMT associated with increased N-cadherin, Snail, and reduced E-cadherin. Overall, these studies demonstrate that Akt(1) suppression in advanced cancers will promote EMT and metastasis.

Although a plethora of information from the cellular and pre-clinical studies have demonstrated the dual role of Akt1 activity in cancer, a correlation between Akt1 activity suppression and promotion metastasis has not been demonstrated in any type of human cancers. Our initial analysis of the cBioportal database revealed the existence of missense mutations in the Akt isoforms that did not modulate its activity thus indicating that genetic mutations in Akt isoforms did not contribute to the onset or aggressiveness of PCa. The MSKCC genomic data showed alterations in the PI3K/Akt pathway in 26% of the advanced stage PCa patient population linking to reduced disease-free survival [22]. Interestingly, 14% of these alterations were in the PTEN gene that is known to contribute to the hyperactivation of the PI3K/Akt pathway [50, 51]. Interestingly, there were no significant differences in the mean mRNA expression levels of Akt isoforms between the altered and unaltered groups. The Trento/Broad/Cornell genomic data on human neuroendocrine PCa [26], however, had 66% of the patients exhibiting alterations in genes from the PI3K/Akt pathway. Once again, 31% of these were as a result of PTEN deletion or amplification. Gene amplifications were also observed in the Akt isoforms (25%, 18% and 31% in Akt1, Akt2, and Akt3, respectively). Fred Hutchinson genome analysis of the human metastatic PCa [25] revealed approximately 81% of the genes from the PI3K/Akt pathway with genetic alterations. While 44% of genetic alterations were observed in PTEN deletion or loss of function mutations, only 4-7% alterations were noted in the Akt isoforms. Exome sequencing by the Broad/Cornell group on the human prostate adenocarcinoma [23] showed only 15% of the patients with genetic alterations in the PI3K/Akt pathway, out of which 7% alterations were in PTEN and a single case of missense mutations was observed in Akt1 and Akt3 isoforms. Genomic data of metastatic PCa from the MSKCC study [27] showed approximately 78% of the patients showing alterations in the PI3K/Akt pathway genes, with the majority of 42% of the alterations found as the PTEN gene deletion, fusion or loss of function mutations. Interestingly, none of these studies showed any significant differences in the mean mRNA expression levels of Akt isoforms between the altered and un-altered groups, suggesting that while genetic alterations in PTEN may have contributed to Akt hyperactivation, there was no evidence on the direct effect of genomic alterations in the Akt1 isoforms on their activity in these PCa samples.

The TCGA data was the sole source of information that allowed comparison of genomic, proteomic expressions and activating phosphorylations of Akt isoforms in a large collection of primary prostate adenocarcinoma samples [24]. Although approximately 51% of the patients showed genetic alterations in genes from the PI3K/Akt pathway, only isolated cases of amplification or deletion in Akt1 (1.4%), Akt2 (1%) and Akt3 (2%) genes were noted. Interestingly, despite the amplification of the Akt isoform mRNAs, no significant differences in their expression between the altered and un-altered groups were observed. These studies revealed that although mutations and deletions in PTEN gene lead to PCa, the lack of difference in mRNA and protein expression (data not shown for Akt2 and Akt3) in the Akt isoforms between the altered and un-altered group indicated that the inhibition of PTEN does not contribute further to the already increased Akt in PCa. Intriguingly, further analysis of S473 and T308 activating phosphorylation of Akt1 showed a reduction in their activity in the altered compared un-altered group. This was corroborated with increased gene expression of EMT markers such as TGFβ1, CDH2 and Snail correlating with higher Gleason score and/or metastatic tumor colonies in sites other than the tumors in prostate. Similarly, a significant correlation between reduced Akt phosphorylation (reduced activity) and higher Gleason score was also observed in TCGA analysis indicating Akt de-addiction contributing to cancer aggressiveness.

Activation of PI3K and Akt as a result of PTEN inactivation has been demonstrated to be a contributing factor for PCa oncogenesis [50, 51]. However, as has been critically reviewed by Blanco-Aparicio *et al*, further cancer progression due to PTEN loss occurs as a result of Akt-independent mechanisms [40]. Several mouse models have revealed that Akt activation, although important for oncogenic transformation, alone is not sufficient for tumorigenesis. Expression of myrAkt1 (active) in the prostate [41] or PTEN^−/−^ mice [42] lead to prostatic inter-epithelial neoplasia and tumor, but not metastasis.

Expression of myrakt1 did not promote breast cancer metastasis in P53^−/−^ mice either [43, 44]. A recent review states that having more Akt in cancer is not always better [39]. While the previous reports from the preclinical studies and the most recent reports from the transgenic mouse models demonstrate increased metastasis in the breast, liver, prostate, and lung (NSCLC) cancers with Akt1 suppression, the cBioportal analysis provides reasonable, if not complete evidence indicating the existence of such a phenomenon in human PCa patients as well. Nevertheless, our data from the human PCa cell lines on the effect of MK-2206 in promoting EMT along with the observation of reduced phosphorylated S473Akt in 5+5 Gleason PCa samples compared to 3+3 Gleason PCa samples, a trend toward elevation of TGFβ1, N-cadherin and Snail mRNA levels in the distant metastatic tumors compared to PCa tumors from MSKCC/UMICH study and reduced phosphorylated S473Akt and T308Akt associated with increased TGFβ1 and N-cadherin mRNA levels in the TCGA study suggest a negative correlation between Akt activity and EMT/metastasis in human the advanced PCa. Recent Phase I/II clinical studies have also reported no significant benefits of using MK-2206 for metastatic cancers [52-57]. However, a large-scale analysis of Akt activity in PCa samples will be needed to further confirm this observation.

## MATERIAL AND METHODS

### Genotyping of TRAMP mice

Genotyping of *TRAMP* (C57BL/6) transgenic mice (Jackson, Bar Harbor, ME) was performed as described [13]. All experiments were carried out in accordance with guidelines set by VA Medical Center in Augusta and as approved by the institutional animal care and use committee. DNA was extracted from the tails of 10- to 21-day old litters (Qiagen, Valencia, CA). *TRAMP* transgene (600bp) was detected by PCR with an annealing temperature of 55°C (forward: 5'-GCGCTGCTGACTTTCTAAACATAAG-3' and reverse: 5'-GAGCTCACGTTAAGTTTTGATGTGT-3'). The internal positive control produced a 324bp fragment (forward: 5'-CTAGGCCACAGAATTGAAAGATCT-3' and reverse: 5'-GTAGGTGGAAATTCAGCATCATCC-3'). *TRAMP* mouse prostates were collected at 12, 24, 32 and 40 weeks and subjected to Western blot analysis.

### Cell lines, reagents, and antibodies

Human PC3 and DU145 cells were obtained from ATCC (Manassas, VA). Cells were maintained in DMEM-G (Hyclone, Logan, UT) with 10% FBS (Atlanta Biologicals, GA), 100 U/ml penicillin, and 100 μg/ml streptomycin in a humidified incubator at 37°C and 5% CO2, and routinely passaged when 80–90% confluent. Antibodies for N-cadherin, TGFβ1, Akt1, pS473Akt, panAkt1 TGFβ-RI and Snail1 were purchased from Cell Signaling (Danvers, MA). Anti-β-actin was purchased from Sigma (St. Louis, MO). Akt inhibitor MK2206 was purchased from Selleckchem (Houston, TX).

### Western blot analysis and immunohistochemistry analysis

Western blot analysis was performed as described previously [58, 59]. Images were scanned at 600dpi, cropped, contrast/brightness adjusted equally across the entire blot and presented without combining any two or more different blots. Densitometry analysis was performed using the NIH Image J Software. Unedited images are provided in Supplemental Figures 2 and 3. Slides containing benign prostatic hyperplasia (BPH), 3+3 Gleason score and 5+5 Gleason score PCa patient sections were subjected for immunohistochemistry using pS473Akt antibodies and counterstained by hematoxylin as described previously [60, 61].

### shRNA-mediated gene silencing and generation of stably silenced PCa cells

Human PC3 and DU145 cells were transfected with SMARTvector 2.0 Lentivirus ShAkt1 or non-targeting ShControl particles (GE Dharmacon, Lafayette, CO). Lentiviral infections were performed in 6 well plates. Lentiviral particles were mixed with 1ml SFM4 Transfx-293 (GE Hyclone, Lafayette, CO) solution and applied to PC3 and DU145 cells with 10 μg polybrene (American bioanalytical, MA). After 16 hours, the medium was replaced with complete EBM-2. After 3 days, GFP was detected using a confocal imaging microscope (LSM510, Carl Zeiss, Germany). Stable silencing of Akt1 as compared to ShControl cells was achieved by puromycin selection (8 μg/ml, Thermo, Grand Island, NY). Post selection, cells were maintained in complete DMEM high glucose medium with 0.6 μg/ml puromycin.

### Analysis of clinical trials on Akt1 inhibitor (MK-2206)

Data from phase 3 clinical trials on the use of Akt inhibitor MK-2206 in the treatment of various cancers were collected from www.clinicaltrials.gov. The basis of the evaluation of MK-2206 efficacy is either the standard therapy or experts' opinions unless otherwise stated. All the single group studies were compared to the first line (standard) therapy from the published literature.

### Gene expression and alteration analysis from patient databases

Information regarding protein and mRNA expression and other genetic alterations in Akt pathway molecules were obtained from the publically available cBioportal http://www.cbioportal.org [20, 21]. Before analyzing genomic alterations in the studies of interest, certain genomic profiles that are mutations and copy number alterations were selected for comparative analysis. The studies of interest are metastatic PCa SU2C/PCF Dream team [27], neuroendocrine PCa [26], castration-resistant PCa [25] and two prostate adenocarcinoma studies [22, 23]. “Protein expression” analysis was performed from the only study available in the database [24]. For the genes of interest, we chose the user-defined option of “PI3K-Akt-mTOR pathway”. Genomic analysis was performed using the OncoPrint option to summarize the genomic alterations of Akt1 from 5 different studies stated above. On the table, rows represented genes and columns represented samples. Genomic alterations including mutations, CNA (amplifications and deletions), and changes in gene expression were analyzed.

### Statistical Analysis

All the data are presented as mean ± SD and were calculated from multiple experiments performed in quadruplicates. For the data analyses, Student's two-tailed t-test or one-way ANOVA were used to determine significant differences between treatment and control groups using the GraphPad Prism 4.03 software and SPSS 17.0 software. All the existing statistical analysis data were obtained from the cBioportal. Data with *P<0.05* were considered significant.

## SUPPLEMENTARY MATERIALS TABLES AND FIGURES



## ABBREVIATIONS

SAF-1: serum amyloid A-activating factor-1
VEGF: vascular endothelial growth factor
miR-125b: micro RNA-125b
CTRL RNA: negative control RNA
UTR: un-translated region
siRNA: short interfering RNA
RT-PCR: reverse transcription polymerase chain reaction
qRT-PCR: quantitative RT-PCR
EMSA: electrophoretic mobility shift assay
PAGE: polyacrylamide gel electrophoresis
CAT: chloramphenicol acetyl transferase
