# Supplementary material for: Genome atlas analysis based profiling of Akt pathway genes in the early and advanced human prostate cancer
Source: Oncoscience. 2019 Jul 2;6(5-6):317–36. doi: 10.18632/oncoscience.482 (PMC6650170; doi:10.18632/oncoscience.482)
Supplement: Supplementary file 1 [file oncoscience-06-317-s001.pdf]

SUPPLEMENTARY MATERIALS

Supplementary Table 1. Data on clinical trials on Akt inhibitor MK-2206 for various cancers collected from www.clinicaltrials.gov

Clinical trial ID	Cancer type	Size	Treatment groups	Results	Compared to First line /Standard therapy
NCT01283035	Recurrent and platinum-resistant ovarian, fallopian tube, or primary peritoneal cancer	5	Single group; MK-2206 monotherapy PO QW for 4 weeks (one cycle); repeat if patient is benefiting	Not available	
NCT01283035	Recurrent and platinum-resistant ovarian, fallopian tube, or primary peritoneal cancer	5	Single group; MK-2206 monotherapy PO QW for 4 weeks (one cycle); repeat if patient is benefiting	Not available	
NCT01349933	Recurrent or metastatic head and neck cancer	21	Single group; MK-2206 monotherapy 200 mg PO QW for 4 weeks (one cycle); repeat if patient is benefiting	OS:10 months (95% CI; 5.9 - 20); PFS: 3.5 months (95% CI; 0.9 - 7.3)	Cisplatin + Gemcitabine; OS: 29.1 months; PFS: 7.0 months [1]
NCT01253447	Relapsed or refractory acute myeloid leukemia (AML)	19	Single group; MK-2206 monotherapy 200 mg PO QW for 4 weeks (one cycle); repeat if patient is benefiting	Study was terminated due to insufficient drug efficacy	
NCT01802320	Previously treated, metastatic or locally advanced colon or rectal cancer	18	Single group; MK-2206 monotherapy PO QW for 4 weeks (one cycle); repeat if patient is benefiting	Not available	
NCT01186705	Metastatic KRAS-Wild-Type, PIK3CA-Mutated, Colorectal Cancer	1	Single group; MK-2206 monotherapy 200 mg PO QW for 4 weeks (one cycle); repeat if patient is benefiting	Not available	
NCT01277757	Advanced breast cancer with a PIK3CA mutation, or an AKT mutation and/or PTEN loss/ PTEN mutation	28	Single group; MK-2206 monotherapy 200 mg PO QW for 4 weeks (one cycle); repeat if patient is benefiting	Not available	
NCT01260701	Advanced Gastric/ Gastro-esophageal Junction Cancer	70	Single group; MK-2206 monotherapy 60 mg PO QOD on days 1-28 (one cycle); repeat if patient is benefiting	OS: 5.1 months (95% CI; 3.7 - 9.4); PFS: 1.8 months (95% CI; 1.7 - 1.8)	Cisplatin + 5-FU; OS: 33 weeks; PFS: 27 week [2]

Clinical trial ID	Cancer type	Size	Treatment groups	Results	Compared to First line /Standard therapy
NCT01169649	Metastatic Neuroendocrine Tumors (NET)	8	Single group; MK-2206 monotherapy 200 mg PO QW for 4 weeks (one cycle); repeat if patient is benefiting	Not available	
NCT01294306	Advanced non-small cell lung cancer progressed after previous response to Erlotinib Hydrochloride therapy	80 (45 EGFR-Mutation and 34 EGFR-WT)	Single group; MK-2206 monotherapy PO QOD on days 1-28; repeat if patient is benefiting	OS: EGFR-Mutated: 10.6 months (95% CI; 8.6 - 23.2); EGFR WT: 11.1 months (95% CI; 7.3 - 22.1);<br>PFS: EGFR-Mutated: 4.4 months (95% CI; 2.7 - 6.6); EGFR WT: 4.6 months (95% CI; 2.9 - 8.5)	Cisplatin + Gemcitabine or Cisplatin + Docetaxel; OS: 19·5 months; PFS: 5.2 months [3]
NCT01519427	Stage III/IV melanoma that failed prior therapy with Vemurafenib or Dabrafenib	2	Single group; MK-2206 monotherapy PO QW for 4 weeks + selumetinib PO BID on days 1-21 (one cycle); repeat if patient is benefiting	OS: 153 days, range (117 to 189);<br>PFS: 105 days, range (42 to 168)	Vemurafenib compared to Decarbazine; OS: 13·6 months; PFS: 6·9 months [4]; Dabrafenib compared to Decarbazine: OS: favoring Dabrafenib; PFS: 5.1 months [5]
NCT01658943	Previously treated metastatic pancreatic cancer	121 (62 mFOLFOX and 58 MK + Selumetinib)	Two groups:<br>1st group: mFOLFOX regimen PO QD on days 1-28;<br>2nd group: MK2206 + selumetinib, MK2206 (135 mg) PO QW and selumetinib PO QD on days 1-28	1st group: OS: 6.7 months (95% CI; 6 - 8.3);<br>FBS: 2 months (95% CI; 1.8 - 2.9);<br>2nd group: OS: 3.9 months (95% CI; 3.5 - 4.6);<br>FBS: 1.9 months (95% CI; 1.8 - 2.1)	
NCT01239355	Previously treated advanced liver cancer	15	Single group; MK-2206 monotherapy 200 mg PO QW for 4 weeks (one cycle); repeat if patient is benefiting	OS: 6.1 months (95% CI; 3.0 - 8.4);<br>PFS: 1.7 months (95% CI; 1.6 - 3.0)	Sorafenib; OS: 10.7 months; PFS: 4.1 months [6]

Clinical trial ID	Cancer type	Size	Treatment groups	Results	Compared to First line /Standard therapy
NCT01466868	Relapsed or refractory diffuse large B Cell Lymphoma (AKTIL)	22	Single group; MK-2206 monotherapy 200 mg PO QW for 4 weeks (one cycle); repeat if patient is benefiting	Not available	
NCT01481129	Relapsed or refractory diffuse large B Cell Lymphoma (AKTIL)	22	Single group; MK-2206 monotherapy PO QW for 4 weeks (one cycle); repeat if patient is benefiting	OS: 9.6 months (95% CI; 2.8 to 18.8); PFS: 1.71 months (95% CI; 0.8 to 1.8)	Gemcitabine + Dexamethasone + Cisplatin; OS: 8.9 months; FBS: 3.1 months [7]
NCT01307631	Recurrent or advanced endometrial cancer	37 (9 PIK3CA Mutant and 28 PIK3CA WT)	Two groups; All with MK-2206 monotherapy PO QW for 4 weeks (one cycle); repeat if patient is benefiting	OS: 8 months; range (0-12 months) PFS: PIK3CA Mutant: 1.6 months (90% CI; 0 - 1.6); PIK3CA WT: 1.8 months (90%CI; 0 - 1.8)	Carboplatin + Paclitaxel; OS: 37 months; PFS: 13 months (Miller DS, Filiaci G, Mannel R, et al. Randomized Phase III Noninferiority Trial of First Line Chemotherapy for Metastatic or Recurrent Endometrial Carcinoma: A Gynecologic Oncology Group Study. LBA2. Presented at the 2012 Society of Gynecologic Oncology Annual Meeting, Austin, TX.)
NCT01481129	Relapsed or refractory diffuse large B Cell Lymphoma (AKTIL)	22	Single group; MK-2206 monotherapy PO QW for 4 weeks (one cycle); repeat if patient is benefiting	OS: 9.6 months (95% CI; 2.8 to 18.8); PFS: 1.71 months (95% CI; 0.8 to 1.8)	Gemcitabine + Dexamethasone + Cisplatin; OS: 8.9 months; FBS: 3.1 months [7]
NCT01258998	Relapsed or refractory lymphoma	59	Single group; MK-2206 monotherapy 200 mg PO QW for 4 weeks (one cycle); repeat if patient is benefiting	OS: Not available PFS: 2.8 months	Gemcitabine + Dexamethasone + Cisplatin; OS: 8.9 months; FBS: 3.1 months [7]. (Cancer. 2004 Oct 15;101(8):1835-42)

Clinical trial ID	Cancer type	Size	Treatment groups	Results	Compared to First line /Standard therapy
NCT01604772	Progressive, recurrent, or metastatic adenoid cyst carcinoma	14	Single group; MK-2206 monotherapy 150 mg PO QW for 4 weeks (one cycle); repeat if patient is benefiting	OS: Not available; PFS: 9.2 months (95% CI; 3.8-11)	No optimal treatment due to inadequate clinical trials. (Uptodate.com; Malignant salivary gland tumors: Treatment of recurrent and metastatic disease)
NCT01333475	Advanced colorectal carcinoma	21 (12 in the 1st group (TAC1) and 9 in the 2nd group (TAC1A))	Two groups:<br>1st group (TAC1) : MK-2206 + AZD6244, MK-2206 90 mg PO QW and AZD6244 Hydrogen sulfate 75 mg PO QD (one cycle); repeat if patient is benefiting<br>2nd group (TAC1A): MK-2206 + AZD6244, MK-2206 135 mg PO QW and AZD6244 Hydrogen sulfate 100 mg PO QD (one cycle); repeat if patient is benefiting	This biomarker-driven phase 2 study was conducted to determine the antitumor activity of dual therapy. OS and PFS are not available	
NCT01425879	Advanced refractory biliary cancer that cannot be removed by surgery	8	Single group; MK-2206 monotherapy 200 mg PO QW for 4 weeks (one cycle); repeat if patient is benefiting	OS: 3.5 months (95% CI; 2.2 - 6.7); PFS: 1.7 months (95% CI; 0.5 - 5.6)	Cisplatin + Gemcitabine; OS: 11.7 months; PFS: 8 months [8]
NCT01239342	Refractory kidney cancer	43 (29 in the MK group and 14 in the Everolimus group) For PFS analysis, 1 patient was excluded from each group	Two groups:<br>1st group: MK-2206 200 mg PO QW for 4 weeks; repeat if patient is benefiting<br>2nd group: Everolimus 10 mg PO QD for 4 weeks; repeat if patient is benefiting	MK-2206 group:<br>OS:<br>23.5 months (95% CI; 10.7 - 37.4)<br>PFS: 3.68 months (95% CI; 1.77 - 5.75)<br>Everolimus group:<br>OS: 15.7 months (95% CI; 6.5 - not estimable)<br>PFS: 5.98 months (95% CI; 5.08 to not estimable)	

Clinical trial ID	Cancer type	Size	Treatment groups	Results	Compared to First line /Standard therapy
NCT01251861	Patients with previously treated prostate cancer	104	Two groups:<br>1st group: Observation on weeks 1-12, then bicalutamide PO QD on weeks 13-44. Patients with a PSA decline of >= 50% may continue on bicalutamide until week 72 in the absence of disease progression or unacceptable toxicity.<br>2nd group: MK2206 PO QW on weeks 1-44 and bicalutamide PO QD on weeks 13-44. Patients with a PSA decline of >= 50% may continue on Akt inhibitor MK2206 and bicalutamide until week 72 in the absence of disease progression or unacceptable toxicity	Not available	
NCT01370070	Recurrent and metastatic nasopharyngeal carcinoma	21	Single group; MK-2206 monotherapy 200 mg PO QW for 4 weeks (one cycle); repeat if patient is benefiting	OS: 10 months (95% CI; 5.9 to 20.0)<br>PFS: 3.5 months (95% CI; 0.9 to 7.3)	Gemcitabine + Cisplatin; OS: 29.1 months; PFS: 7 months [9]

Abbreviations: OS (median), overall survival; PFS (median), progression-free survival; PO, by mouth; QW, every week; QOD, every other day; QD, every day; WT, wild-type

References:

[1] J.C. Lin, J.S. Jan, C.Y. Hsu, W.M. Liang, R.S. Jiang, W.Y. Wang, Phase III study of concurrent chemoradiotherapy versus radiotherapy alone for advanced nasopharyngeal carcinoma: positive effect on overall and progression-free survival, J Clin Oncol, 21 (2003) 631-637.
[2] H. Bleiberg, T. Conroy, B. Paillot, A.J. Lacave, G. Blijham, J.H. Jacob, L. Bedenne, M. Namer, P. De Besi, F. Gay, L. Collette, T. Sahmoud, Randomised phase II study of cisplatin and 5-fluorouracil (5-FU) versus cisplatin alone in advanced squamous cell oesophageal cancer, Eur J Cancer, 33 (1997) 1216-1220.
[3] R. Rosell, E. Carcereny, R. Gervais, A. Vergnenegre, B. Massuti, E. Felip, R. Palmero, R. Garcia-Gomez, C. Pallares, J.M. Sanchez, R. Porta, M. Cobo, P. Garrido, F. Longo, T. Moran, A. Insa, F. De Marinis, R. Corre, I. Bover, A. Illiano, E. Dansin, J. de Castro, M. Milella, N. Reguart, G. Altavilla, U. Jimenez, M. Provencio, M.A. Moreno, J. Terrasa, J. Munoz-Langa, J. Valdivia, D. Isla, M. Domine, O.

Molinier, J. Mazieres, N. Baize, R. Garcia-Campelo, G. Robinet, D. Rodriguez-Abreu, G. Lopez-Vivanco, V. Gebbia, L. Ferrera-Delgado, P. Bombaron, R. Bernabe, A. Bearz, A. Artal, E. Cortesi, C. Rolfo, M. Sanchez-Ronco, A. Drozdowskyj, C. Queralt, I. de Aguirre, J.L. Ramirez, J.J. Sanchez, M.A. Molina, M. Taron, L. Paz-Ares, P.-C. Spanish Lung Cancer Group in collaboration with Groupe Francais de, T. Associazione Italiana Oncologia, Erlotinib versus standard chemotherapy as first-line treatment for European patients with advanced EGFR mutation-positive non-small-cell lung cancer (EURTAC): a multicentre, open-label, randomised phase 3 trial, Lancet Oncol, 13 (2012) 239-246.

[4] G.A. McArthur, P.B. Chapman, C. Robert, J. Larkin, J.B. Haanen, R. Dummer, A. Ribas, D. Hogg, O. Hamid, P.A. Ascierto, C. Garbe, A. Testori, M. Maio, P. Lorigan, C. Lebbe, T. Jouary, D. Schadendorf, S.J. O'Day, J.M. Kirkwood, A.M. Eggermont, B. Dreno, J.A. Sosman, K.T. Flaherty, M. Yin, I. Caro, S. Cheng, K. Trunzer, A. Hauschild, Safety and efficacy of vemurafenib in BRAF(V600E) and BRAF(V600K) mutation-positive melanoma (BRIM-3): extended follow-up of a phase 3, randomised, open-label study, Lancet Oncol, 15 (2014) 323-332.

[5] A. Hauschild, J.J. Grob, L.V. Demidov, T. Jouary, R. Gutzmer, M. Millward, P. Rutkowski, C.U. Blank, W.H. Miller, Jr., E. Kaempgen, S. Martin-Algarra, B. Karaszewska, C. Mauch, V. Chiarion-Sileni, A.M. Martin, S. Swann, P. Haney, B. Mirakhur, M.E. Guckert, V. Goodman, P.B. Chapman, Dabrafenib in BRAF-mutated metastatic melanoma: a multicentre, open-label, phase 3 randomised controlled trial, Lancet, 380 (2012) 358-365.

[6] J.M. Llovet, S. Ricci, V. Mazzaferro, P. Hilgard, E. Gane, J.F. Blanc, A.C. de Oliveira, A. Santoro, J.L. Raoul, A. Forner, M. Schwartz, C. Porta, S. Zeuzem, L. Bolondi, T.F. Greten, P.R. Galle, J.F. Seitz, I. Borbath, D. Haussinger, T. Giannaris, M. Shan, M. Moscovici, D. Voliotis, J. Bruix, S.I.S. Group, Sorafenib in advanced hepatocellular carcinoma, N Engl J Med, 359 (2008) 378-390.

[7] M. Crump, T. Baetz, S. Couban, A. Belch, D. Marcellus, K. Howson-Jan, K. Imrie, R. Myers, G. Adams, K. Ding, N. Paul, L. Shepherd, J. Iglesias, R. Meyer, Gemcitabine, dexamethasone, and cisplatin in patients with recurrent or refractory aggressive histology B-cell non-Hodgkin lymphoma: a Phase II study by the National Cancer Institute of Canada Clinical Trials Group (NCIC-CTG), Cancer, 101 (2004) 1835-1842.

[8] J. Valle, H. Wasan, D.H. Palmer, D. Cunningham, A. Anthoney, A. Maraveyas, S. Madhusudan, T. Iveson, S. Hughes, S.P. Pereira, M. Roughton, J. Bridgewater, A.B.C.T. Investigators, Cisplatin plus gemcitabine versus gemcitabine for biliary tract cancer, N Engl J Med, 362 (2010) 1273-1281.

[9] L. Zhang, Y. Huang, S. Hong, Y. Yang, G. Yu, J. Jia, P. Peng, X. Wu, Q. Lin, X. Xi, J. Peng, M. Xu, D. Chen, X. Lu, R. Wang, X. Cao, X. Chen, Z. Lin, J. Xiong, Q. Lin, C. Xie, Z. Li, J. Pan, J. Li, S. Wu, Y. Lian, Q. Yang, C. Zhao, Gemcitabine plus cisplatin versus fluorouracil plus cisplatin in recurrent or metastatic nasopharyngeal carcinoma: a multicentre, randomised, open-label, phase 3 trial, Lancet, 388 (2016) 1883-1892.

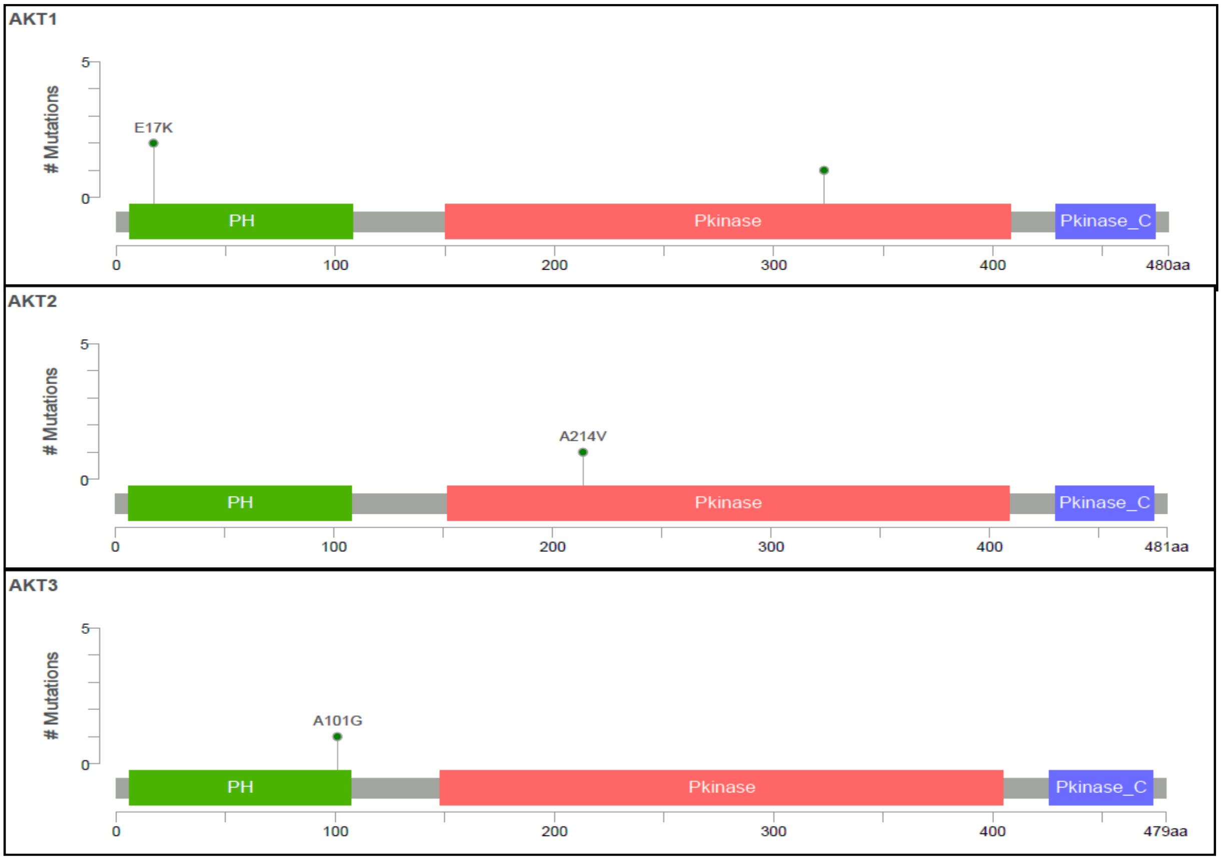

Supplemental Figure 1: Mutation in the Akt isoforms based on the data collected from the cBioportal database. This Figure shows the mutations detected in the Akt1, Akt2 and Akt3 isoforms in PCa patient samples based on the 6 genomic analysis studies available in the cBioportal database.

Images from Figure 8A

PC3

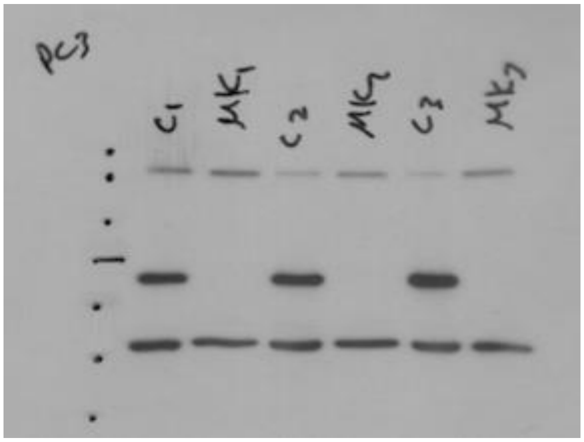

DU145

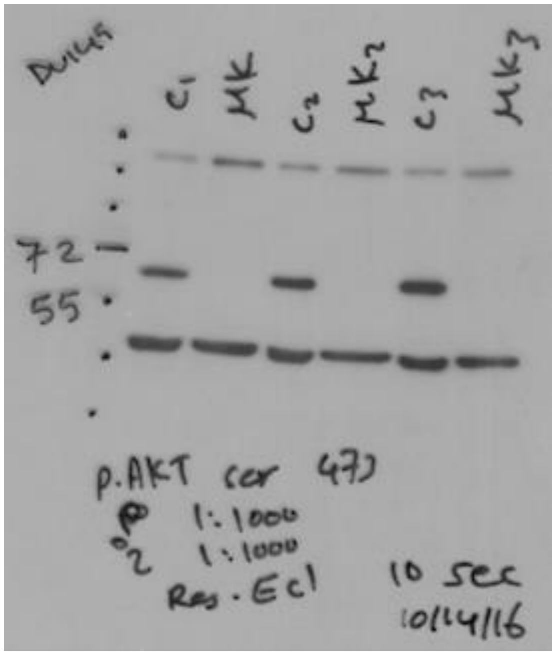

N-Cadherin

pSer473Akt

β-actin

Images from Figure 8C

PC3

Snail

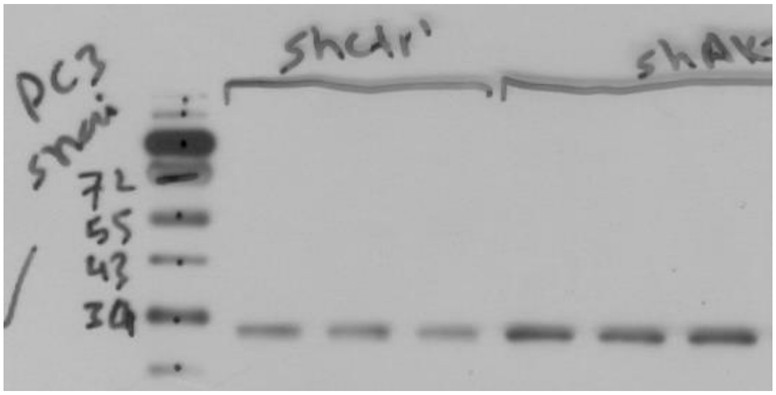

βActin

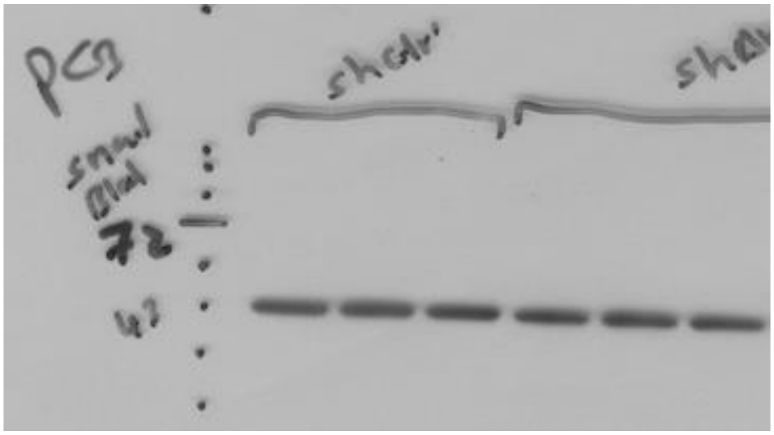

Supplemental Figure 2: Original Western blot images

Images from Figure 8C

PC3

TGFβ-R1

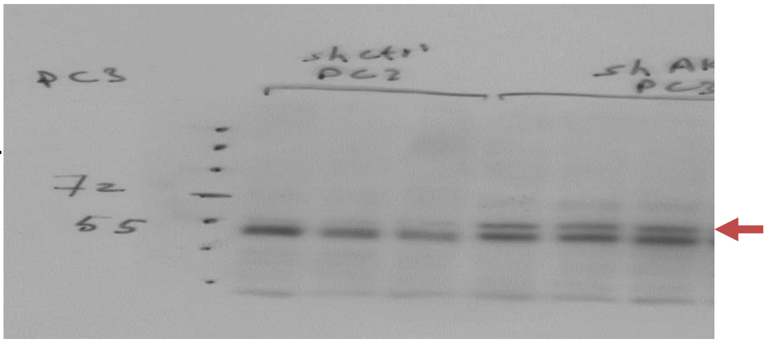

βActin

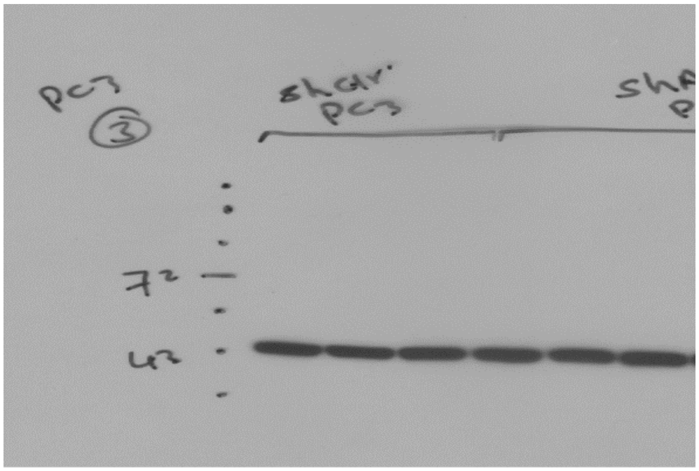

DU145

Snail

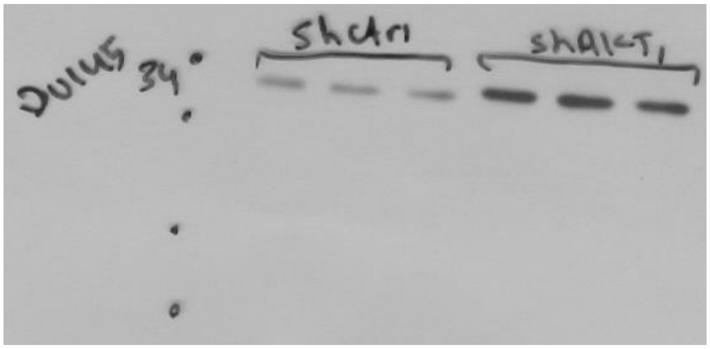

βActin

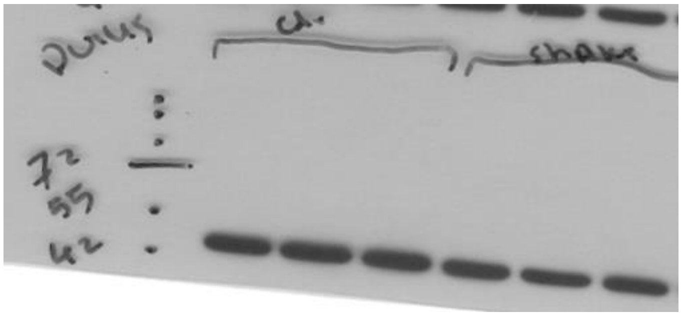

Supplemental Figure 3: Original Western blot images

Images from Figure 8C

DU145

TGFβ-R1

βActin

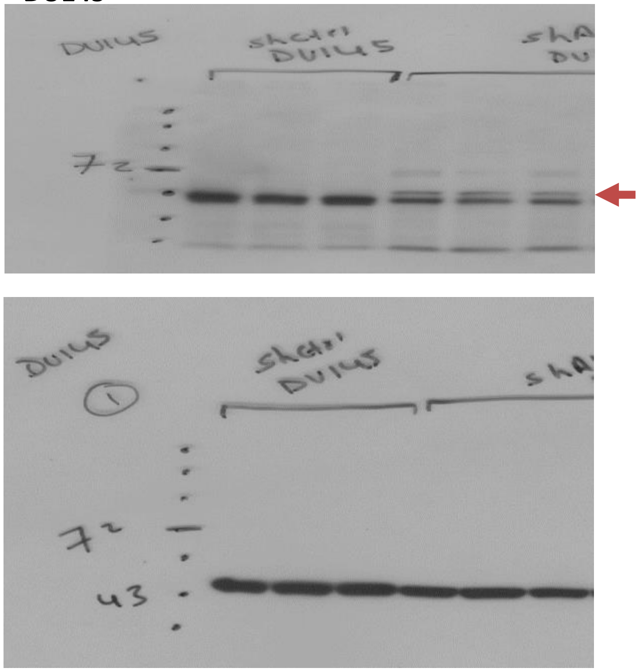

Images from Figure 8E

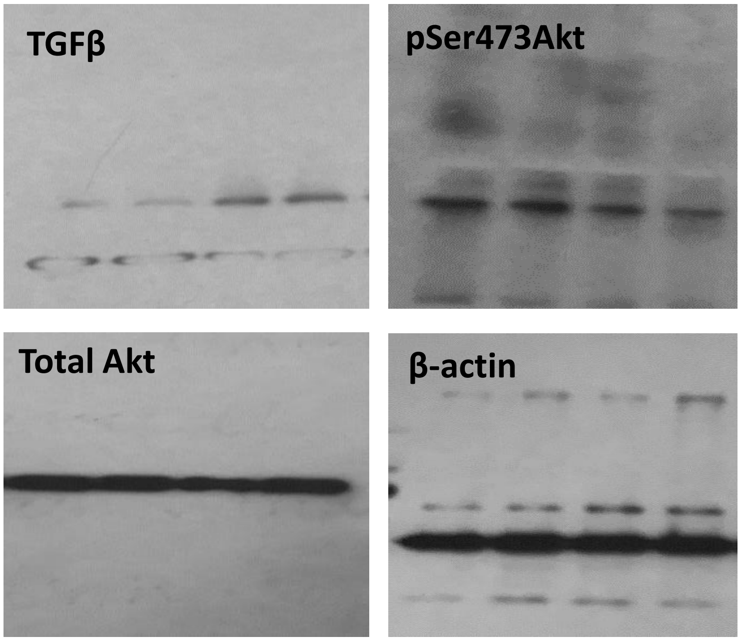

Supplemental Figure 4: Original Western blot images
